# Flapless Mini Implants Immediately Loaded for Full‐Arch Overdenture Rehabilitation: An Up‐To‐15‐Year Retrospective Study

**DOI:** 10.1111/clr.70017

**Published:** 2025-08-07

**Authors:** Gerardo Pellegrino, Zoran Zaccheroni, Subhi Tayeb, Andrea Oliverio, Edoardo Mancuso, Lorenzo Bonifazi, Amerigo Giudice, Carlo Barausse, Pietro Felice

**Affiliations:** ^1^ Unit of Oral Surgery, Department of Biomedical and Neuromotor Sciences University of Bologna Bologna Italy; ^2^ Department of Health Sciences Magna Graecia University of Catanzaro Catanzaro Italy; ^3^ Private Practice Imola Italy; ^4^ Prosthetic Unit, Department of Biomedical and Neuromotor Sciences University of Bologna Bologna Italy

**Keywords:** dental implants, marginal bone loss, mini dental implants, overdentures, retrospective study

## Abstract

**Objectives:**

This study aimed to evaluate the survival rates of Mini Dental Implants (MDIs) placed in both the maxilla and the mandible, and their associated overdenture prostheses in edentulous patients over short‐, medium‐, and long‐term follow‐up periods.

**Materials and Methods:**

Patients rehabilitated with Mini Dental Implants (diameter ≤ 2.9 mm) as support for overdenture prostheses with a minimum follow‐up period of 3 years were included in the study. Data from eligible patients were collected, and marginal bone loss (MBL) was assessed for each implant. The primary outcomes for the prostheses and implants included failure rates, complications, and implant‐related MBL.

**Results:**

The study analyzed 83 patients and 334 implants over an 8.09 ± 3.96‐year mean follow‐up. The cumulative implant survival rate at 15 years was 86.3% (95% CI: 79.5%–91.0%). Mean MBL increased from 0.09 ± 0.44 mm at implant placement to 1.79 ± 0.82 mm at 15 years, with Lodi Biomax implants exhibiting significantly lower annual bone loss compared to Dentatus Atlas implants (*p* < 0.001). The prosthetic survival rate was 95.45% (95% CI: 91.1–99.81). Longer follow‐up (*p* = 0.018) and fewer adjustments (*p* < 0.001) reduced risks of complication occurrence. Additionally, Lodi Biomax implants had fewer complications compared to Dentatus Atlas (*p* = 0.039).

**Conclusion:**

Mini Dental Implants with a diameter between 2.4 and 3 mm showed high survival rates over follow‐ups of up to 15 years. Their use offers a viable prosthetic solution for edentulous patients, minimizing surgical invasiveness, rehabilitation time, and costs, particularly when a fixed prosthesis is either not feasible or not requested by the patient.

## 1 Introduction

Bone crest resorption begins immediately after tooth extraction and is most pronounced during the first year, with up to 60% of the alveolar ridge width being lost. Resorption is chronic and progressive in both the maxilla and the mandible, particularly in the anterior regions where buccolingual resorption leads to the formation of a narrow or “knife‐edge” alveolar ridge.

This condition presents a significant challenge for rehabilitating complex cases with standard‐diameter implants (Marcello‐Machado et al. 2018).

The conventional approach to rehabilitate horizontally atrophic ridges is to reconstruct the bone to allow a standard implant placement; however, regenerative techniques usually come with high costs, long rehabilitative times, and possible complications, particularly in full‐arch cases.

A possible alternative is represented by narrow‐diameter implants, measuring between 3 mm and 3.5 mm. Recent studies have shown that narrow implants are adequate as retainers for mandibular overdentures, comparable to standard‐diameter implants (El‐Sheikh et al. 2012).

Mini dental implants (MDIs), with diameters ranging from 1.8 mm to 2.9 mm, have been explored as a minimally invasive option in specific clinical scenarios due to the smaller size and reduced costs.

Their use may be beneficial for patients with systemic health challenges or anatomical limitations (Srinivasan et al. 2023), such as severe bone resorption or reduced interdental space. MDIs are commonly employed to stabilize overdentures, offering a less invasive and cost‐effective alternative to standard implants. Typically designed as one‐piece units with spherical or retentive attachments such as Locator systems, MDIs are often placed using flapless techniques that, according to Leles et al. this type of surgery requires less clinical time and allows for easier intraoral prosthetic incorporation of attachments compared to open‐flap surgeries (Leles et al. 2022; Leles, de Oliveira Moura‐Neto, et al. 2024). Immediate loading is common, particularly in edentulous arches where multiple implants (usually four) are used to anchor mandibular overdentures, as reported by Zygogiannis et al. (2017) According to Group 1 of the 2018 ITI Consensus Conference (Jung et al. 2018), the potential advantages of using MDI include ensuring adequate tooth‐implant and implant‐implant distances in sites with reduced mesiodistal width, reducing the need for or complexity of lateral bone augmentation procedures to minimize morbidity, allowing for simultaneous rather than multi‐stage bone augmentation procedures, and providing greater prosthetic flexibility in certain clinical situations. In the classification by Klein, Schiegnitz, and Al‐Nawas (Klein et al. 2014) narrow‐diameter implants (NDI) are divided into three categories: Category 1 includes implants with a diameter of less than 2.5 mm (“Mini‐implants”), Category 2 includes implants with a diameter ranging from 2.5 mm to less than 3.3 mm, and Category 3 includes implants with a diameter between 3.3 mm and 3.5 mm. According to the guidelines provided by the 2018 ITI Consensus, implants belonging to Categories 1 and 2, classified as MDI, may be considered for the support of definitive complete mandibular overdentures, while only Category 2 implants can be used for the replacement of a single tooth in the anterior region with reduced interdental width.

However, mini‐implants and narrow‐diameter implants should be used cautiously. Reducing the implant diameter could increase the risk of fractures due to their reduced mechanical strength. The implant neck is a potential fracture zone when subjected to high flexural forces (Akça et al. 2003). As highlighted by studies on the stress distribution mechanism on the implant and/or bone, the biomechanical stress exerted by MDI's is significantly higher compared to standard implants (Patil et al. 2021; Pisani et al. 2018). Finite element analysis (FEA) in literature has been used to evaluate the stress distribution patterns generated in the components associated with implant overdentures. Meijer et al. (1992) investigated stress distribution by using a 2D model of the mandible with 2 implants by applying a vertical load of 100 N. Jofre (2010) carried out FEA and clinical trials evaluating marginal bone loss with 2 splinted versus 2 unsplinted mini‐implant–retained overdentures at the 2‐year follow‐up. The FEA showed the minimum principal stress (−118 MPa) in the bone surrounding the unsplinted mini implants compared with that of the splinted implants (−56.8 MPa).

Due to these mechanical limitations, mini‐implants and narrow‐diameter implants are recommended primarily for enhancing the retention and stability of mandibular overdentures in cases of limited bone thickness. A direct advantage of these implants lies in their simplified and minimally invasive surgical techniques, leading to shorter treatment and recovery times and reduced costs. This makes rehabilitation accessible to patients who cannot undergo more invasive and extensive surgical procedures.

Several reviews have already showed the predictability of rehabilitation with mini‐implants or narrow‐diameter implants, focusing on survival rates, surgical techniques (flap or flapless), and the influence of implant length on success and survival rates (Marcello‐Machado et al. 2018). Moreover, these reviews describe success and survival rates independently of the type of prosthesis used for prosthetic rehabilitation.

Previously reported survival rates from systematic reviews for implants with diameters < 3.5 mm were 90% (9‐year follow‐up), 88% (12‐year follow‐up), and 75% (8‐year follow‐up for implants < 3.3 mm) (Klein et al. 2014; Sohrabi et al. 2012; Alshenaiber et al. 2023; Park et al. 2023; Majid 2024). A meta‐analysis comparing the survival rates of standard‐diameter implants and narrow‐diameter implants found no statistically significant differences in implant survival (Ortega‐Oller et al. 2014).

However, currently limited information exists in the literature regarding the indications and prognosis of mini‐implants. Therefore, it could be important to expand the understanding of the clinical performance indicators for these overdenture retention implants.

## 2 Materials and Methods

The study design was a retrospective, single‐cohort observational study conducted at the Oral Surgery Unit of the Dental Clinic, University of Bologna. Based on the clinical rationale and the existing evidence, the null hypothesis of the present study was that implant‐specific characteristics—such as implant diameter, length, or system—would not significantly affect long‐term implant survival in edentulous patients treated with flapless, immediately loaded mini implants supporting overdentures. Clinical and radiographic records, both paper‐based and digital, of patients treated from January 2009 to July 2024, were analyzed. Patients who could not be contacted or were unwilling to participate in the study, and consequently did not sign specific informed consent, were excluded. The study adhered to the principles outlined in the Declaration of Helsinki for clinical research involving human subjects and received favorable approval from the Local Ethics Committee (protocol no. 544/2024). The study followed the STROBE cohort reporting guidelines.

### 2.1 Inclusion Criteria

The study included patients with horizontally atrophic jaws rehabilitated with MDIs (diameter ≤ 3 mm) supporting overdenture prostheses, with a minimum of 3 years of radiographic documentation. Patients with less than 3 years of follow‐up were excluded from statistical analysis unless implant failure occurred within this timeframe.

### 2.2 Data Collection and Classification

All patient records were initially reviewed and assessed for eligibility based on predefined criteria. Relevant patient information, including age (at the time of implant placement), sex, and whether rehabilitation was performed in the maxilla or mandible, was recorded. For each patient, data on the number of implants, their positions, diameters, and lengths were documented. For the prosthetic components, the frequency of relinings for prosthesis adaptation, retention system replacements, adjustments, and modifications for proper maintenance were also recorded.

### 2.3 Outcome Measures

The primary outcome of the study was the survival rate of MDIs. Secondary outcomes included prosthetic survival, implant and prosthetic complications, implant and prosthetic failure, peri‐implant marginal bone loss (MBL), the need for prosthetic component modifications, and retention system replacements.

Implant survival was defined as the maintenance of the implant in situ with the absence of implant mobility, pathological bleeding on probing, and patient‐reported pain during clinical examinations. Peri‐implantitis was defined, according to the consensus report of Group 4 of the 2017 World Workshop on the Classification of Periodontal and Peri‐Implant Diseases and Conditions, as a plaque‐associated pathological condition occurring in the tissues around dental implants, characterized by peri‐implant mucosal inflammation and subsequent progressive loss of supporting bone (Berglundh et al. 2018).

**FIGURE 1:**
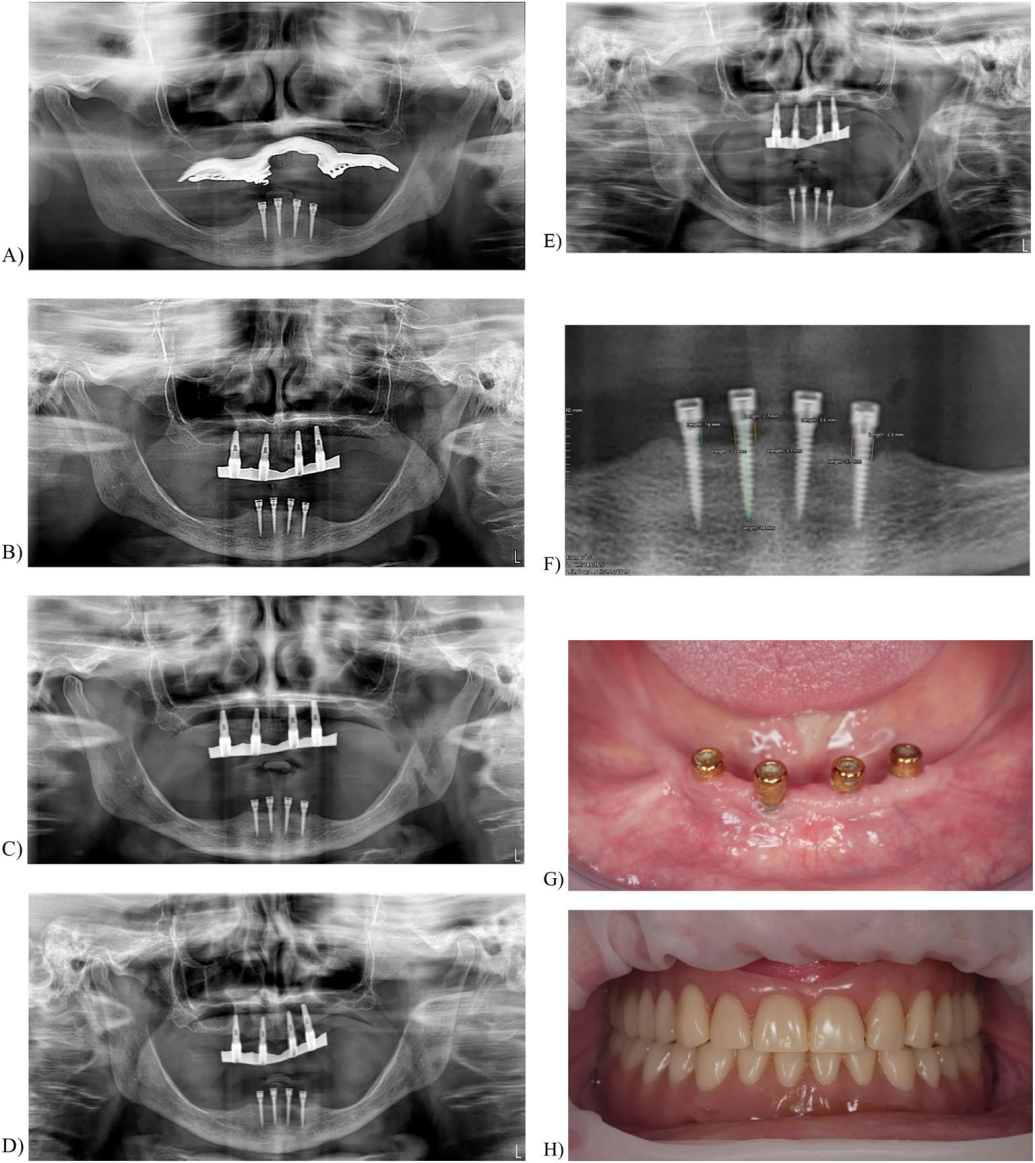
Example of a edentulous patient treated with four Biomax Lodi Locator Implant System. (A) Orthopantomography (OPG) showing the T0. (B) Orthopantomography (OPG) showing the T1. (C) Orthopantomography (OPG) showing the T5 (D) Orthopantomography (OPG) showing the T8 (E) Orthopantomography (OPG) showing the T10 (F) MBL calibration with Weasis Software (G, H) Clinical results.

**FIGURE 2:**
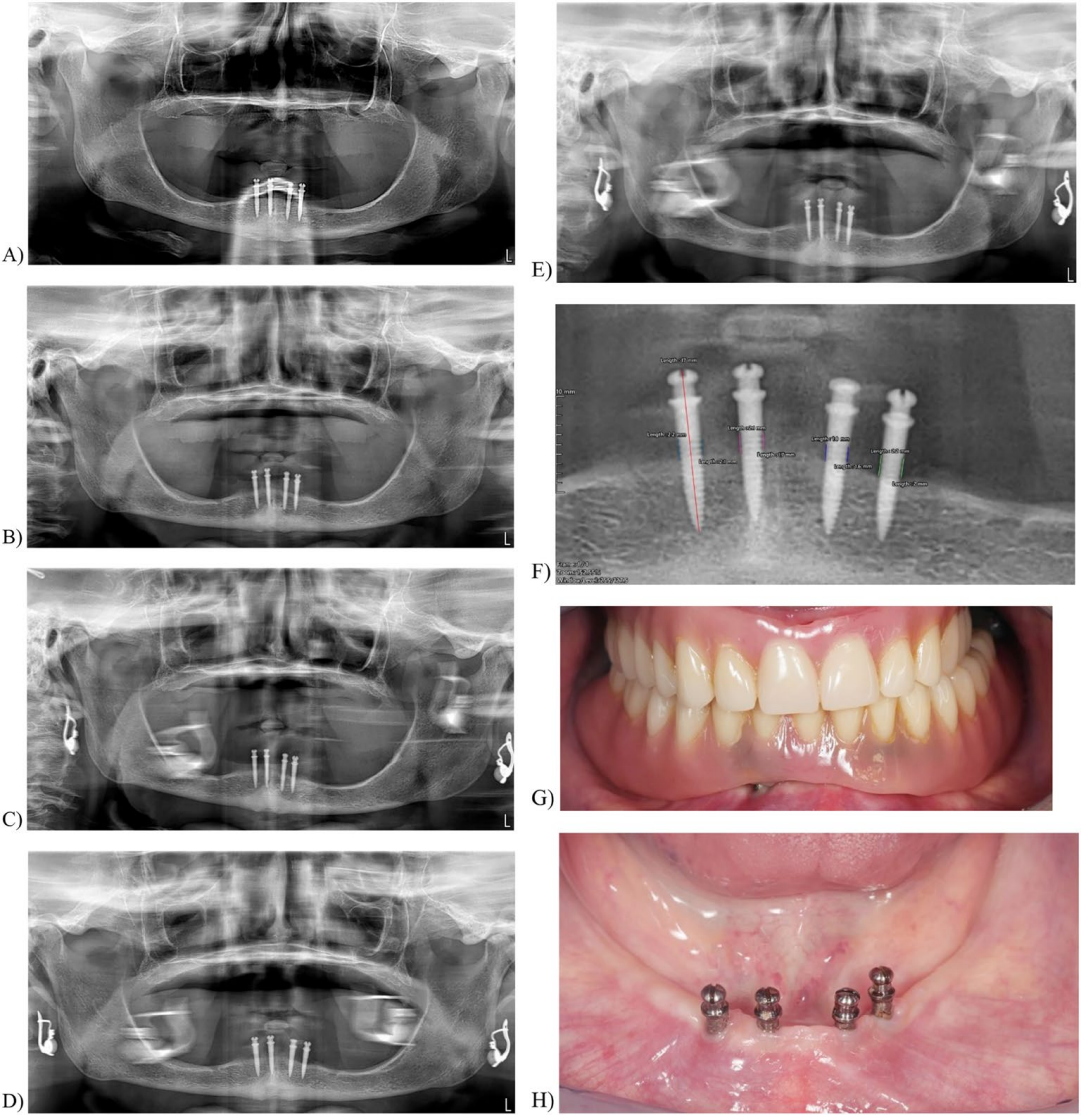
Example of a edentulous patient treated with four Dentatus Atlas Implant System. (A) Orthopantomography (OPG) showing the T0. (B) Orthopantomography (OPG) showing the T1. (C) Orthopantomography (OPG) showing the T5 (D) Orthopantomography (OPG) showing the T8 (E) Orthopantomography (OPG) showing the T10 (F) MBL calibration with Weasis Software (G, H) Clinical results.

Peri‐implant marginal bone level (MBL) changes were assessed using orthopantomographs. Measurements were taken at implant placement (T0) and at 1, 3, 5, 8, and 10 years post‐loading (T1, T3, T5, T8, and T10) according to the follow‐up of every patient. The orthopantomographs were acquired using the NewTom GiANO radiological system (NewTom, CEFLA, Italy), with an average exposure time of 13 s per patient. For all patients, an 18 × 24 cm field of view was used. The Weasis software (Weasis DICOM medical viewer) was employed for measurements, which were calibrated for each image using the known implant parameters such as length or diameter (Figure 1F and Figure 2F). Measurements of the mesial and distal bone crest levels adjacent to each implant were recorded to the nearest 0.01 mm and averaged at the implant, patient, and group levels. Measurements were performed parallel to the implant axis. The reference points for the linear measurements were the most coronal margin of the implant collar and the most coronal, radiographically appreciable point of bone‐to‐implant contact. The annual MBL rate was calculated by dividing the difference between the MBL measurement at the final follow‐up radiograph and baseline (T0) by the number of follow‐up years.

All bone loss measurements were performed on the same device by an experienced clinician and subsequently reviewed and validated by a second operator with equivalent clinical experience to confirm compliance with the previously described guidelines. Prosthetic failure was defined as the necessity to replace the prosthesis delivered at implant loading with a new one due to its inability to be adjusted chairside. Complications were divided into biological (e.g., peri‐implant diseases) and prosthetic categories. The classification of prosthetic complications was adapted from previously established methodologies (Ravidà et al. 2019; Naert et al. 2004), and included both mechanical issues (e.g., fractures, chipping, attachment failures) and soft tissue complications (e.g., mucositis, soreness, and decubitus ulcers) affecting the denture‐bearing areas. The success rate was assessed at the implant level, with cases classified as successful when no biological or biomechanical complications were observed during the follow‐up period (Elsyad 2012).

### 2.4 Statistical Analysis

Descriptive statistics were used to summarize the data. Categorical variables were reported as absolute and relative frequencies, while continuous variables were described using means and standard deviations. The normal distribution of the variables was assessed using the Kolmogorov–Smirnov test, while homoscedasticity was evaluated using Levene's test. For the analysis of implant failure, a mixed‐effects Weibull proportional hazards regression model was employed. Fixed effects included demographic, implant‐specific, and clinical variables, while random effects accounted for inter‐patient variability. A sample size calculation indicated that 311 implants would be required to detect a hazard ratio of 3 between implant diameter groups with 80% power, a 5% significance level, and an event rate of 8.38%. Given the actual sample size (334 implants, 28 failures), the post hoc power was estimated at 82.8%. For marginal bone loss (MBL), a mixed‐effects linear regression model was used, fitted with the restricted maximum likelihood (REML) estimation method. Fixed effects included patient demographics, implant‐specific variables, follow‐up duration, jaw type, and the presence of complications, while random effects accounted for intra‐patient variability. The occurrence of multiple prosthetic complications was analyzed using the Andersen‐Gill extension of the Cox proportional hazards model. Cluster‐robust standard errors were applied to account for correlations arising from multiple prostheses and multiple events within the same patient. Model adequacy was confirmed through stable convergence of the estimation procedure and a highly significant Wald chi‐square test (*p* < 0.0001), indicating the contribution of covariates to explaining the hazard of complications. Model assumptions were verified and confirmed for all models. For the Weibull and Cox regressions, proportionality and time‐to‐event assumptions were deemed acceptable, while residual analysis for the linear model showed no major deviation from normality or homoscedasticity. Sample size calculations were performed for the prosthetic complication model. For a hazard ratio of 3.0 and an event rate of 31.8%, 82 prostheses and 27 events would be required to achieve 80% power (*α* = 0.05). For a hazard ratio of 2.5, the required sample increased to 118 prostheses and 38 events. Statistical significance for all tests was set at *p* < 0.05. Data were analyzed using Statistical Software Stata 18 (StataCorp. 2023, Release 18. College Station, TX: StataCorp LLC).

## 3 Results

The study sample included 83 patients who had previously received MDI ≤ 2.9 mm. Among these patients, 52 were female (62.65%) and 31 were male (37.35%), with a mean age of 79.72 ± 9.48 years, ranging from 59 to 100 years. On average, 4.02 implants were placed per patient, all in native bone (Table 1).

**TABLE 1:** Demographic overview at patient level.

*N*. Patients	83
Age (years)	79.72 ± 9.48
Gender	
Male	31 (37.35%)
Female	52 (62.65%)
Type of implant system	
Biomax Lodi	181 (54.19%)
Dentatus atlas	153 (45.81%)
Implant diameter	2.59 ± 0.25 mm
Implant leght	12.36 ± 1.72 mm
Type of jaw	
Maxilla	45 (13.45%)
Mandible	289 (86.53%)

### 3.1 Implants Outcomes

The total number of implants included in the study was 334. Two types of implants were observed among the patients: 181 (54.19%) Biomax Lodi Locator Implant System (Figure 1) and 153 (45.81%) Dentatus Atlas Implant System (Figure 2). Of the total implants, 289 (86.53%) were placed in the mandible, whereas 45 (13.47%) were placed in the maxilla.

Among the included patients, the mean diameter of the MDI was 2.59 ± 0.25 mm, with a minimum diameter of 2.2 mm and a maximum of 2.9 mm. Additionally, the implants had an average length of 12.36 ± 2.59 mm, ranging from a minimum of 10 mm to a maximum of 17 mm. All implants supported overdenture prostheses.

**GRAPH 1:**
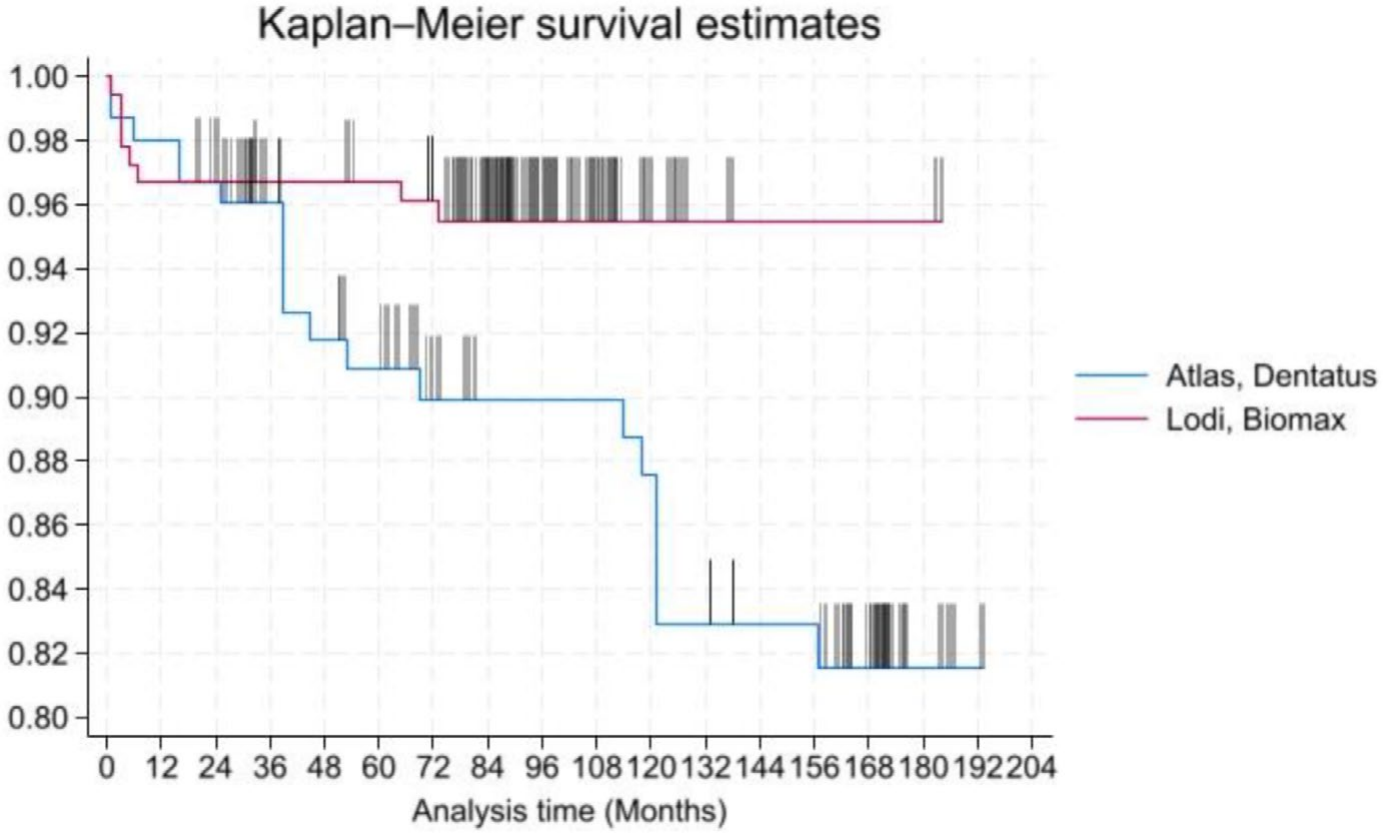
Kaplan–Meier survival estimate. Time (in months) is represented on the x‐axis, while cumulative survival is depicted on the y‐axis. Vertical tick marks are present to indicate censoring.

**GRAPH 2:**
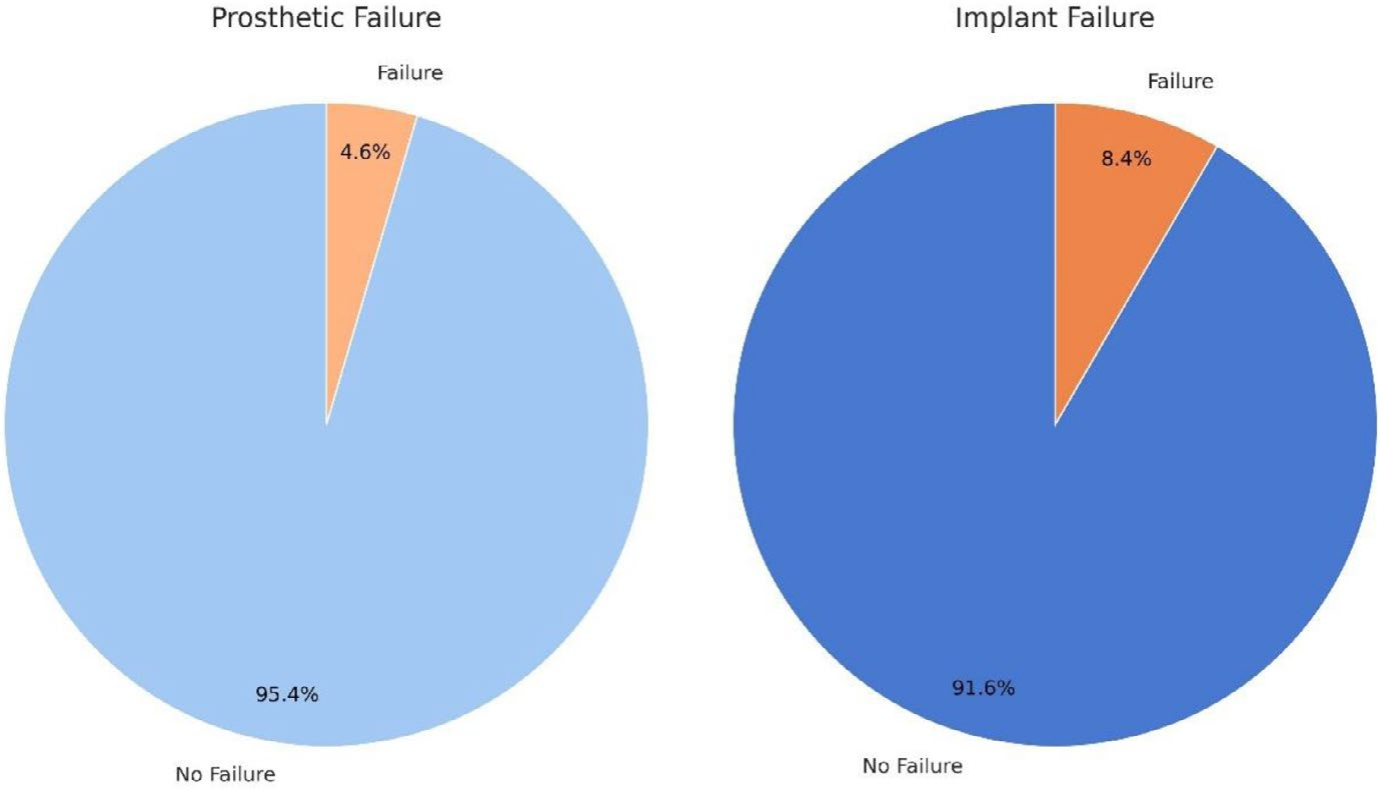
Pie charts displaying the crude failure rates of mini dental implants and full‐arch overdentures. Implant failures occurred in 28 out of 334 implants (8.38%), while prosthetic failures occurred in 4 out of 88 overdentures (4.55%). These rates are descriptive and do not account for follow‐up duration or censored data.

The mean follow‐up period for the implants was 8.09 ± 3.96 years. The cumulative implant failure rate at 15 years was 13.7% (95% CI: 9.0%–20.5%). Specifically, the 15‐year cumulative implant failure rate estimated by Kaplan–Meier analysis was 18.4% for Dentatus Atlas implants (95% CI: 12.1–27.5) and 4.5% for Lodi Biomax implants (95% CI: 2.3–8.8) (Graph 1). The cumulative implant survival rate at 15 years, as estimated by Kaplan–Meier analysis, was 86.3% (95% CI: 79.5%–91.0%) (Graph 2).

Among the included patients, 96.71% (323/334, 95% CI: 94.79–98.62) experienced no implant‐related complications. Of the 3.29% who did encounter complications, only cases of mucositis were diagnosed. During the follow‐up period, five cases of peri‐implantitis were reported (1.5%, 5/334, 95% CI: 0.19–2.8).

Regarding implant‐specific characteristics, implants with a diameter below 2.5 mm showed a significantly increased risk of failure, with a hazard ratio (HR) of 55.6 (95% CI: 3.70–835.9; *p* = 0.004), indicating that wider implants (≥ 2.5 mm) were associated with a notably lower risk of failure. Conversely, implant length was not significantly associated with implant survival (HR = 1.06; *p* = 0.779). In contrast, longer follow‐up duration was linked to a significantly reduced risk of implant failure (HR = 0.92; *p* < 0.001). No statistically significant associations were observed for implant type, jaw location (maxilla vs. mandible), patient age, or the presence of complications (all *p* > 0.05). Specifically, the comparison between Dentatus Atlas and Lodi Biomax implants did not reveal a significant difference in failure risk (*p* = 0.738). Similarly, maxillary implants were not associated with a significantly higher failure risk compared to mandibular implants (*p* = 0.932). The presence of complications was also not identified as a significant risk factor for implant failure (*p* = 0.563). Nevertheless, a higher failure rate of 18.18% was observed in cases with complications, compared to 8.05% in cases without complications (Table 2).

**TABLE 2:** Results of the mixed‐effects Weibull proportional hazards regression model analyzing factors associated with implant failure. Hazard ratios (HR) represent the relative risk of failure for each variable. Values below 1 indicate a protective effect, while values above 1 indicate increased risk. Fixed effects include demographic, implant‐specific, and clinical variables, while random effects account for inter‐patient variability. Statistically significant associations (*p* < 0.05) are highlighted, with implant diameter and total follow‐up duration showing significant protective effects against failure.

Implant failure among different groups							
_t	Failure rate (%)—(falied/non failed)	Haz. ratio	Std. err.	*z*	*P* > |z|	[95% conf. interval]	
Age		0.9672611	0.0465225	−0.69	0.498	0.8802446	1.06288
Sex							
Female	9.62 (20/208)	—	—	—	—	—	—
Male	6.35 (8/126)	0.7441442	0.7648059	−0.29	0.774	. 0992707	5.578184
Implant diameter							
≥ 2.5 mm		—	—	—	—	—	—
< 2.5 mm		55.64578	76.92517	2.91	0.004**	3.704456	835.8724
Implant length		1.063002	0.2314294	0.28	0.779	0.693771	1.62874
Total follow‐up (months)		0.9186772	0.015225	−5.12	0.000***	0.8893161	0.9490077
Implant type							
Atlas	13.07 (20/153)	—	—	—	—	—	—
Lodi Biomax	4.42 (8/181)	. 718,359	. 7,116,253	−0.33	0.738	0.1030668	5.006846
Jaw type							
Mandible	7.96 (23/289)	—	—	—	—	—	—
Maxilla	11.11 (5/45)	1.119605	1.489823	0.08	0.932	0.0824895	15.19607
Complications							
No	8.05 (26/323)	—	—	—	—	—	—
Yes	18.18 (2/11)	1.717258	1.606999	0.58	0.563	0.2743383	10.74941
_cons		0.0004938	0.0022474	−1.67	0.094	6.59e‐08	3.697184
/ln_p		0.7361161	0.1776404			0.3879474	1.084285
patientID							
var(_cons)		6.381139	3.644553			2.083239	19.54598

*Note:* Statistically significant values are in bold; *p* value codes: * *p* < 0.05; ** *p* < 0.01; *** *p* < 0.001.

**GRAPH 3:**
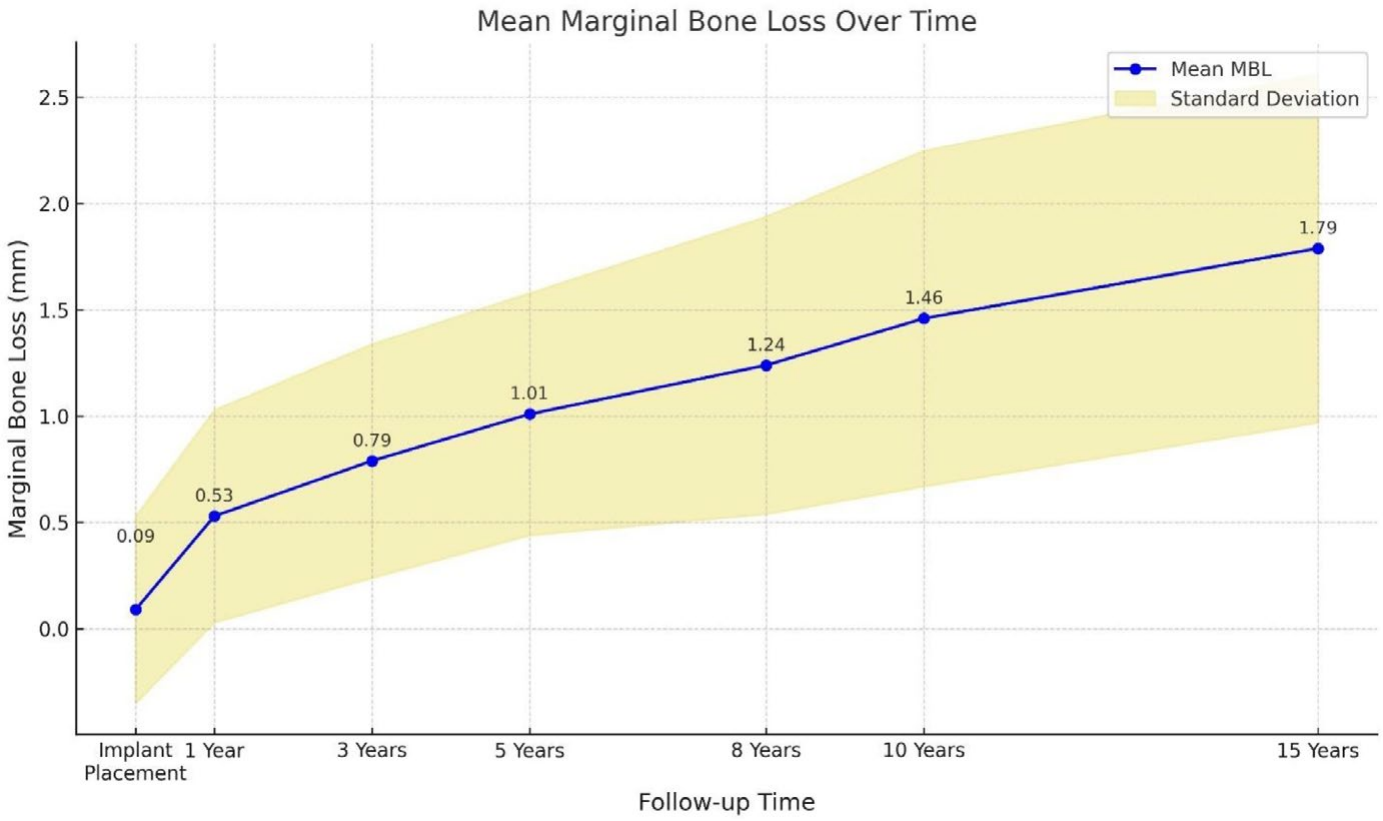
Mean Marginal Bone Loss (MBL) in the different timeframes (Baseline, 1, 3, 5, 8, 10 years).

The progression of marginal bone loss (MBL) was evaluated at various follow‐up intervals, showing a gradual increase over time. At implant placement, the mean MBL was minimal at 0.09 ± 0.44 mm, indicating negligible bone loss shortly after implant placement. At the 1‐year follow‐up, the mean MBL increased to 0.53 ± 0.50 mm, reflecting the early phase of bone remodelling typically observed after implantation. By the 3‐year follow‐up, the mean MBL reached 0.79 ± 0.55 mm, continuing to increase to 1.01 ± 0.57 mm at 5 years. At the eighth year of follow‐up, the mean MBL was 1.25 ± 0.70 mm, with a further increase to 1.46 ± 0.79 mm at 10 years. The 15‐year follow‐up recorded the highest mean MBL value of 1.79 ± 0.82 mm (Graph 3).

The results indicated that the average annual MBL rate differed between the two implant types. Atlas implants exhibited a mean annual MBL rate of 0.24 ± 0.2 mm, whereas Lodi Biomax implants showed a lower rate of 0.17 ± 0.1 mm. This difference was statistically significant (*p* < 0.001), indicating less bone loss associated with Lodi Biomax implants compared to Atlas implants.

Among other analysed variables, follow‐up duration was significantly associated with MBL suggesting that longer follow‐ups were linked to reduced annual marginal bone loss (*p* < 0.001). In contrast, other variables such as sex, age, implant diameter, implant length, maxillary location, and the presence of complications did not show statistically significant associations with MBL (Table 3).

**TABLE 3:** Presents the results of a mixed‐effects linear regression model designed to analyze factors associated with marginal bone loss (MBL). The model was fitted using the restricted maximum likelihood (REML) estimation method, accounting for both fixed effects and random effects. Fixed effects included patient demographics (e.g., sex, age), implant‐specific variables (e.g., diameter, length, type), follow‐up duration, jaw type, and the presence of complications. Random effects were included to account for intra‐patient variability, with patients treated as random intercept.

Marginal bone loss (MBL) among different groups								
Model	Total (%)	Coefficient	Std. err.	Annual MBL rate	*z*	*P* > |z|	[95% conf. interval]	
(constant)		0.7256305	0.1574759	—	4.61	0.000***	0.4169834	1.034278
Sex				—				
Female	208 (62.28)	—	—	0.2 ± 0.15	—	—	—	—
Male	126 (37.72)	−0.0078177	0.0199282	0.2 ± 0.17	−0.39	0.695	−0.0468763	0.0312408
Age		−0.001217	0.0010881	—	−1.12	0.263	−0.0033496	0.0009156
Implant diameter		−0.0670627	0.0411446	—	−1.63	0.103	−0.1477045	0.0135792
Implant length		0.0009402	0.0052443	—	0.18	0.858	−0.0093385	0.0112188
Total follow‐up (months)		−0.0022465	0.0002203	—	−10.20	0.000***	−0.0026784	−0.0018147
Implant type								
Atlas	153 (45.81)	—	—	0.24 ± 0.2	—	—	—	—
Lodi Biomax	181 (54.19)	−0.0810522	0.0205699	0.17 ± 0.1	−3.49	0.000***	−0.1213686	−0.0407359
Jaw type				—				
Mandible	289 (86.53)	—	—	0.2 ± 0.15	—	—	—	—
Maxilla	45 (13.47)	0.0444401	0.0311695	0.24 ± 0.22	1.43	0.154	−0.0166511	0.1055313
Complications				—				
No	323 (96.71)	—	—	0.2 ± 0.16	—	—	—	—
Yes	11 (3.29)	0.0351739	0.0373388	0.24 ± 0.21	0.94	0.346	−0.0380089	0.1083566

Random‐effects parameters	Estimate	Std. err.	[95% conf. interval]
var(R.patient)	0.0042704	0.0012996	0.0023519–0.0077539
var(Residual)	0.0118126	0.0010903	0.0098578–0.014155

*Note:* Statistically significant values are in bold; *p* values codes: *p < 0.05; **p < 0.01; ***p < 0.001.

### 3.2 Prosthetic Outcomes

**GRAPH 4:**
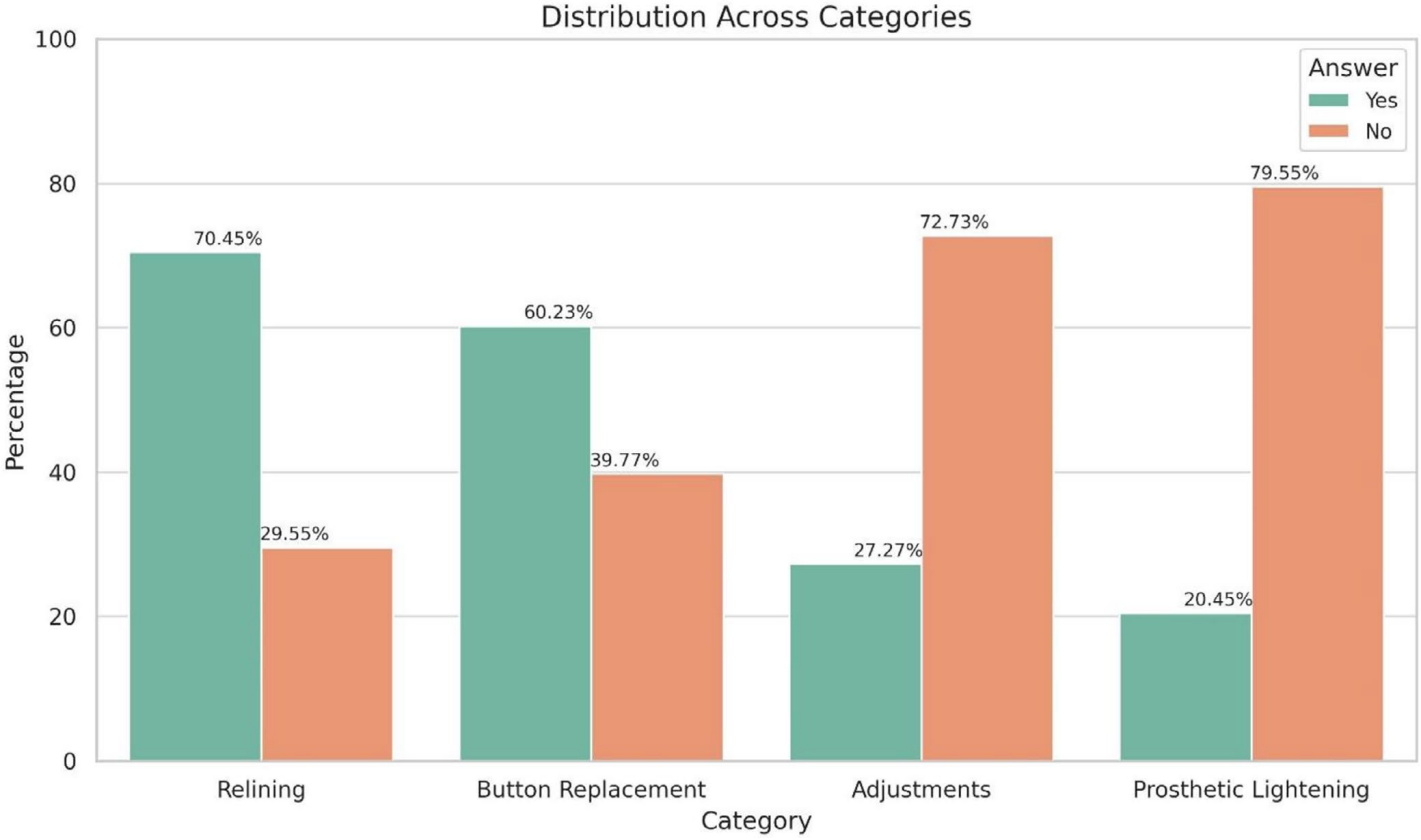
Prosthetic maintenance legend Bar Chart.

A total of 88 overdenture prostheses were included in the study. The crude prosthetic failure rate was 4.55% (4/88, 95% CI: 0.19%–8.9%), while the cumulative prosthetic survival rate estimated by Kaplan–Meier analysis was 95.4% (95% CI: 88.2%–98.3%) over the follow‐up period. Specific parameters related to prosthetic maintenance were evaluated in the study. Relining was required in 70.45% (62/88) of the cases, retention buttons were replaced in 60.23% (53/88), prosthetic adjustments were necessary in 72.73% (64/88), and prosthetic reliefs were performed in 79.55% (70/88) (Graph 4).

**GRAPH 5:**
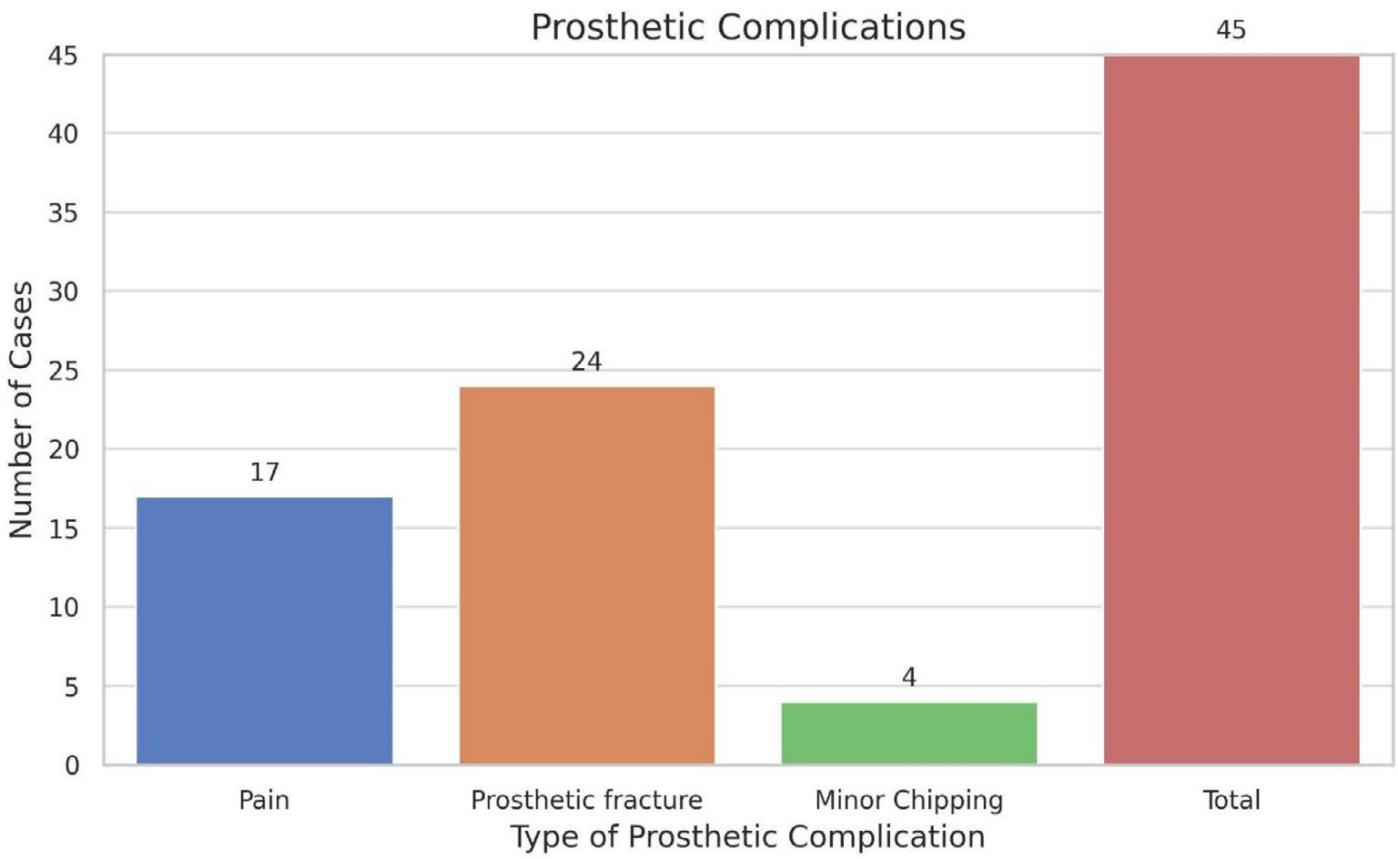
Bar chart illustrating the frequency of prosthetic complications recorded throughout the follow‐up period. Complications were categorized as fractures, minor chipping, and prosthetic pain—the latter defined as discomfort reported by the patient in the absence of clinical signs of biological pathology, and resolved through prosthetic adjustments.

Prosthetic complications included 24 cases of prosthetic fractures and 4 cases of chipping. Additionally, 17 cases of prosthetic pain were reported during the follow‐up period (Graph 5).

Age did not show a statistically significant association with the risk of complications (*p* = 0.134). Male patients appeared to have a 61% higher risk of the event occurring compared to females, but this association was not statistically significant (*p* = 0.163). Implant length showed a marginal effect, with a trend toward reduced complication risk as length increased, presenting an HR of 0.82 (*p* = 0.067).

Follow‐up duration was a significant factor in the model, with an HR of 0.99 (*p* = 0.018), suggesting a slight reduction in the risk of complications over time. Among maintenance‐related factors, the number of prosthetic adjustments emerged as a significant predictor of complication risk, with an HR of 1.32 (*p* < 0.001), indicating an increased risk with a higher number of required adjustments.

No significant associations were found for the number of relinings (*p* = 0.151) or occlusal reductions (*p* = 0.686). Similarly, neither the type of arch (mandibular vs. maxillary) nor the need for attachment replacements showed a significant relationship with the risk of complications (*p* = 0.719 and *p* = 0.223, respectively). Finally, compared to Atlas implants, the model identified a significantly lower HR for Lodi Biomax implants (HR of 0.52; *p* = 0.039), suggesting a reduced risk of complications associated with the latter (Table 4).

**TABLE 4:** The Andersen‐Gill extension of the Cox proportional hazards model was implemented to address the occurrence of multiple prosthetic complications over time within the same patient. To account for the correlation arising from multiple prostheses and multiple events within the same patient, cluster‐robust standard errors were applied at the patient level. Model adequacy was confirmed by stable convergence of the estimation procedure and by a highly significant Wald chi‐square test (*p* < 0.0001), indicating that the included covariates substantially contributed to explaining the hazard of complications.

Prosthetic complications							
			Robust				
_t	Complication rate (%)	Haz. ratio	std. err.	*z*	*P* > |z|	[95% conf. interval]	
Age		1.025746	0.0174149	1.50	0.134	0.9921749	1.060453
Sex							
Female	48.21	—	—	—	—	—	—
Male	56.25	1.611439	0.5507778	1.40	0.163	0.8246685	3.148822
Implant length		0.8181086	0.0895652	−1.83	0.067	0.6601198	1.013909
Implant diameter		1.246837	1.067402	0.26	0.797	0.2328663	6.675945
Follow‐up (months)		0.9914841	0.0035714	−2.37	0.018*	0.9845089	0.9985087
Number of relinings		1.26064	0.2032718	1.44	0.151	0.9190498	1.729191
Number of prosthetic adjustments		1.324216	0.105483	3.53	0.000***	1.132804	1.547971
Number of occlusal reductions		1.157368	0.4179326	0.40	0.686	0.570292	2.348797
Jaw type							
Mandible	53.84	—	—	—	—	—	—
Maxilla	30	0.8163567	0.4601628	−0.36	0.719	0.2704431	2.464246
Attachment replacements							
No	28.57	—	—	—	—	—	—
Yes	66.04	1.608787	0.6279646	1.22	0.223	0.7485925	3.457416
Implant type							
Atlas	60.53	—	—	—	—	—	—
Lodi Biomax	44	0.5207882	0.1650127	−2.06	0.039*	0.2798699	0.9690941

*Note:* Statistically significant values are in bold; *p* values codes: *p < 0.05; **p < 0.01; ***p < 0.001.

## 4 Discussion

The findings of this study reveal that MDIs supporting overdenture prostheses represent a viable therapeutic option, with only 28 failures out of 334 implants placed, resulting in a cumulative survival rate of 86.3% over a follow‐up period of up to 15 years. These data supports the robustness of the therapeutic protocol employed when a fixed rehabilitation is not required by the patient. The 2018 ITI Consensus Report and some systematic reviews reported survival rates ranging from 90.9% to 98% for reduced‐diameter implants with follow‐ups of up to 6 years post‐loading, indicating that our data aligns with, if not exceeds, these benchmarks (Marcello‐Machado et al. 2018; Jung et al. 2018; Klein et al. 2014; Park et al. 2023; Enkling et al. 2019).

The mean age of the cohort, 79 years, suggests that this therapeutic rationale was well accepted by elderly patients.

Both implant systems performed well in this study. While the Biomax Lodi system showed a trend toward a lower failure rate compared to the Dentatus Atlas system, the differences were not statistically significant, suggesting that both systems are reliable options in similar clinical settings.

The variation between the two implant systems could potentially be attributed to differences in the prosthetic connection and its biomechanical behavior, though further investigation is warranted. The average follow‐up period of approximately 8 years allowed for thorough monitoring of implant performance, revealing that longer follow‐up durations were associated with a significantly reduced risk of failure. This finding clinically suggests that failures predominantly occur within the early years of loading. Implant location (mandible or maxilla) did not significantly affect failure risk; however, the small sample size of maxillary mini‐implants may limit the reliability of this finding. Similarly, the limited number of implant failures (*n* = 28) may have affected the precision of some regression estimates, particularly for implant diameter. Although a targeted power and sample size analysis confirmed the adequacy of the sample for detecting a clinically meaningful difference, findings related to this variable should still be interpreted with caution.

Regarding MBL, a gradual increase was observed over time, from minimal values of 0.09 mm at implant placement to more substantial values of 1.79 mm at 15 years. Nevertheless, the annual mean MBL rate was significantly lower for Biomax Lodi implants compared to Dentatus Atlas implants, at 0.17 mm versus 0.24 mm, respectively, illustrating a long‐term clinical advantage for the former. These values are favorable considering the follow‐up period and are consistent with, or superior to, some studies conducted by Zygogiannis et al. (2016) and Curado et al. (2023), a systematic review reporting MBL values ranging from 0.6 mm to 1.43 mm (Schiegnitz and Al‐Nawas 2018) and a previous retrospective study on MDIs that reported an average of 0.70 mm (Anitua et al. 2016). Other variables, such as sex, age, implant length, and location, showed no statistically significant correlations with bone loss, suggesting that implant type and follow‐up duration are central factors in managing MBL.

Concerning implant‐supported overdenture prostheses, the survival rate was remarkably high at 95.45%. Despite the overall success, maintenance was required; most patients underwent relining, prosthetic adjustments, and replacement of retention caps. While prosthetic complications were present in 27.27% of cases, they were generally minor, with fractures and chipping being the most common issues. A noteworthy association was identified between prosthetic adjustments and complication risk, where a greater number of adjustments was a significant predictor of complications, emphasizing the importance of meticulous initial prosthetic planning to minimize subsequent interventions. The adjustment and complication rates were consistent with other studies, highlighting the necessity of not underestimating the maintenance demands of MDIs for overdentures (Mundt et al. 2015; Schwindling and Schwindling 2016; Lemos et al. 2017). Biomax Lodi implants appeared to show greater reliability compared to Dentatus Atlas implants, with a trend toward a reduced complication risk. This suggests the potential effectiveness of a more rigid prosthetic connection. However, the relatively limited number of prosthetic complications observed (*n* = 28) may have constrained the statistical power of the multivariable model, and the potential underpowering should be considered when interpreting the results, particularly regarding moderate effect sizes. Caution is therefore warranted in drawing definitive conclusions from these findings.

MDI treatment may successfully address the challenges faced by elderly patients with dentures, particularly those with a medical history requiring a minimally invasive approach or those undergoing drug therapies such as anticoagulants or bisphosphonates. MDIs, as described by Leles, Curado, et al. (2024), can be effective in improving masticatory performance and bite force in edentulous patients, particularly when compared to rehabilitations with standard removable dentures. Additionally, patients with low income or dental surgery‐related anxiety may also benefit from this treatment approach (Sohrabi et al. 2012). Griffits et al. and Della Vecchia et al. reported that the cost of four MDIs was equivalent to that of a single standard implant (Griffitts et al. 2005; Della Vecchia et al. 2018) or at least to a standard solution requiring two standard implants for acceptable overdenture retention. For this reason, MDI treatment could be considered a suitable rehabilitation option for elderly patients, particularly those already using complete removable prostheses.

The implant cumulative survival rate of 86.3% and prosthetic survival rate of 95.45% over up to 15 years of follow‐up underscore the effectiveness of mini dental implants as a long‐term solution. However, the mechanical limitations of MDIs (Akça et al. 2003) and their lower survival rate compared to standard implants (de Souza et al. 2015) must be considered. Additionally, the limitations of this study, including the evaluation of MBL using orthopantomograms, which are commonly used radiographic tools for follow‐up in such cases, and its retrospective design that presents a significant risk of bias, which may affect data homogeneity and completeness, should be acknowledged. Moreover, the study did not include patient‐reported outcomes (PROs), which are increasingly relevant in implant dentistry. While this was beyond the scope of the present retrospective design, future prospective studies should consider integrating validated PROs to provide a more comprehensive assessment of treatment success.

## 5 Conclusion

The results of this study indicate that Mini Dental Implants with a diameter between 2.4 and 3.0 mm can be a feasible therapeutic option when a fixed prosthesis is not required or indicated for the patient. This kind of rehabilitation exhibits good stability over a follow‐up period of up to 15 years. MDIs, when used as retainers for overdenture prostheses, can represent a valid alternative to standard implants placed in regenerated bone, particularly for edentulous patients with narrow, “knife‐edge” alveolar ridges, offering the advantages of reduced invasiveness, lower costs, and shorter rehabilitation times. However, it is essential to emphasize that every clinical decision should be tailored to the individual case and the patient's specific needs, taking into account the limitations of this surgical choice and the potential alternatives.

## Author Contributions

**Gerardo Pellegrino:** conceptualization, methodology. **Zoran Zaccheroni:** conceptualization, methodology. **Subhi Tayeb:** formal analysis, data curation, writing – original draft. **Andrea Oliverio:** writing – original draft, data curation. **Edoardo Mancuso:** data curation, writing – original draft. **Lorenzo Bonifazi:** writing – original draft. **Amerigo Giudice:** writing – review and editing. **Carlo Barausse:** methodology, supervision. **Pietro Felice:** conceptualization, methodology, validation.

## Conflicts of Interest

The authors declare no conflicts of interest.

## Data Availability

The data that support the findings of this study are available from the corresponding author upon reasonable request.

## References

[clr70017-bib-0001] Akça, K. , M. C. Cehreli , and H. Iplikçioğlu . 2003. “Evaluation of the Mechanical Characteristics of the Implant‐ Abutment Complex of a Reduced‐Diameter Morse‐Taper Implant. A Nonlinear Finite Element Stress Analysis.” Clinical Oral Implants Research 14, no. 4: 444–454. 12869007 10.1034/j.1600-0501.2003.00828.x

[clr70017-bib-0002] Alshenaiber, R. , C. Barclay , and N. Silikas . 2023. “The Effect of Mini Dental Implant Number on Mandibular Overdenture Retention and Attachment Wear.” BioMed Research International 30, no. 2023: 7099761. 10.1155/2023/7099761 PMC10164865 37168235

[clr70017-bib-0003] Anitua, E. , J. Saracho , L. Begoña , and M. H. Alkhraisat . 2016. “Long‐Term Follow‐Up of 2.5‐Mm Narrow‐Diameter Implants Supporting a Fixed Prostheses.” Clinical Implant Dentistry and Related Research 18, no. 4: 769–777. 10.1111/cid.12350. 25913652

[clr70017-bib-0004] Berglundh, T. , G. Armitage , M. G. Araujo , et al. 2018. “Peri‐Implant Diseases and Conditions: Consensus Report of Workgroup 4 of the 2017 World Workshop on the Classification of Periodontal and Peri‐Implant Diseases and Conditions.” Journal of Clinical Periodontology 45, no. Suppl 20: S286–S291. 10.1111/jcpe.12957. 29926491

[clr70017-bib-0005] Curado, T. F. F. , J. R. Silva , L. N. Nascimento , et al. 2023. “Implant Survival/Success and Peri‐Implant Outcomes of Titanium‐Zirconium Mini Implants for Mandibular Overdentures: Results From a 1‐Year Randomized Clinical Trial.” Clinical Oral Implants Research 34, no. 8: 769–782. 10.1111/clr.14102. 37254798

[clr70017-bib-0006] de Souza, R. F. , A. B. Ribeiro , M. P. Della Vecchia , et al. 2015. “Mini vs. Standard Implants for Mandibular Overdentures: A Randomized Trial.” Journal of Dental Research 94, no. 10: 1376–1384. 10.1177/0022034515601959. 26294416

[clr70017-bib-0007] Della Vecchia, M. P. , C. R. Leles , T. R. Cunha , et al. 2018. “Mini‐Implants for Mandibular Overdentures: Cost‐Effectiveness Analysis Alongside a Randomized Trial.” JDR Clinical & Translational Research 3, no. 1: 47–56. 10.1177/2380084417741446. 30938654

[clr70017-bib-0008] El‐Sheikh, A. M. , O. F. Shihabuddin , and S. M. F. Ghoraba . 2012. “Two Versus Three Narrow‐Diameter Implants With Locator Attachments Supporting Mandibular Overdentures: A Two‐Year Prospective Study.” International Journal of Dentistry 2012: 285684. 22754570 10.1155/2012/285684 PMC3382982

[clr70017-bib-0009] Elsyad, M. A. 2012. “Prosthetic Aspects and Patient Satisfaction With Resilient Liner and Clip Attachments for Bar‐ and Implant‐Retained Mandibular Overdentures: A 3‐Year Randomized Clinical Study.” International Journal of Prosthodontics 25: 148–156. 22371836

[clr70017-bib-0010] Enkling, N. , M. Haueter , A. Worni , F. Müller , C. R. Leles , and M. Schimmel . 2019. “A Prospective Cohort Study on Survival and Success of One‐Piece Mini‐Implants With Associated Changes in Oral Function: Five‐Year Outcomes.” Clinical Oral Implants Research 30, no. 6: 570–577. 10.1111/clr.13444. 31021481

[clr70017-bib-0011] Griffitts, T. M. , C. P. Collins , and P. C. Collins . 2005. “Mini Dental Implants: An Adjunct for Retention, Stability, and Comfort for the Edentulous Patient.” Oral Surgery, Oral Medicine, Oral Pathology, Oral Radiology, and Endodontology 100, no. 5: e81–e84. 10.1016/j.tripleo.2005.06.018. 16243233

[clr70017-bib-0012] Jofre, J. 2010. “Effect of Splinting Mini‐Implants on Marginal Bone Loss: A Biomechanical Model and Clinical Randomized Study With Mandibular Overdentures.” International Journal of Oral & Maxillofacial Implants 25, no. 6: 1137–1144. 21197490

[clr70017-bib-0013] Jung, R. E. , B. Al‐Nawas , M. Araujo , et al. 2018. “Group 1 ITI Consensus Report: The Influence of Implant Length and Design and Medications on Clinical and Patient‐Reported Outcomes.” Clinical Oral Implants Research 29, no. Suppl 16: 69–77. 10.1111/clr.13342. 30328189

[clr70017-bib-0014] Klein, M. O. , E. Schiegnitz , and B. Al‐Nawas . 2014. “Systematic Review on Success of Narrow‐Diameter Dental Implants.” International Journal of Oral & Maxillofacial Implants 29, no. Suppl: 43–54. 10.11607/jomi.2014suppl.g1.3. 24660189

[clr70017-bib-0015] Leles, C. R. , T. F. F. Curado , L. N. Nascimento , et al. 2024. “Changes in Masticatory Performance and Bite Force After Treatment With Mandibular Overdentures Retained by Four Titanium‐Zirconium Mini Implants: One‐Year Randomised Clinical Trial.” Journal of Oral Rehabilitation 51, no. 8: 1459–1467. 10.1111/joor.13722. 38685704

[clr70017-bib-0016] Leles, C. R. , L. de Oliveira Moura‐Neto , J. R. Silva , et al. 2024. “A Cross‐Sectional CBCT Assessment of the Relative Position of One‐Piece Titanium‐Zirconium Mini‐Implants Placed for Mandibular Overdentures Using Non‐Guided Surgery.” Clinical Oral Implants Research 35, no. 11: 1475–1484. 10.1111/clr.14335. 39041319

[clr70017-bib-0017] Leles, C. R. , M. S. de Paula , T. F. F. Curado , et al. 2022. “Flapped Versus Flapless Surgery and Delayed Versus Immediate Loading for a Four Mini Implant Mandibular Overdenture: A RCT on Post‐Surgical Symptoms and Short‐Term Clinical Outcomes.” Clinical Oral Implants Research 33, no. 9: 953–964. 35818640 10.1111/clr.13974

[clr70017-bib-0018] Lemos, C. A. , F. R. Verri , V. E. Batista , J. F. Júnior , C. C. Mello , and E. P. Pellizzer . 2017. “Complete Overdentures Retained by Mini Implants: A Systematic Review.” Journal of Dentistry 57: 4–13. 10.1016/j.jdent.2016.11.009. 27888049

[clr70017-bib-0019] Majid, O. W. 2024. “Can Narrow‐Diameter Implants Enhance Patient‐Reported Outcomes for Mandibular Implant‐Retained Overdentures?” Evidence‐Based Dentistry 25, no. 3: 131–133. 10.1038/s41432-024-01017-3. 38745081

[clr70017-bib-0020] Marcello‐Machado, R. M. , F. Faot , A. J. Schuster , G. G. Nascimento , and A. A. del Bel Cury . 2018. “Mini‐Implants and Narrow Diameter Implants as Mandibular Overdenture Retainers: A Systematic Review and Meta‐Analysis of Clinical and Radiographic Outcomes.” Journal of Oral Rehabilitation 45, no. 2: 161–183. 29125652 10.1111/joor.12585

[clr70017-bib-0021] Meijer, H. J. , J. H. Kuiper , F. J. Starmans , and F. Bosman . 1992. “Stress Distribution Around Dental Implants: Influence of Superstructure, Length of Implants, and Height of Mandible.” Journal of Prosthetic Dentistry 68, no. 1: 96–102. 10.1016/0022-3913(92)90293-j. 1403929

[clr70017-bib-0022] Mundt, T. , C. Schwahn , T. Stark , and R. Biffar . 2015. “Clinical Response of Edentulous People Treated With Mini Dental Implants in Nine Dental Practices.” Gerodontology 32, no. 3: 179–187. 10.1111/ger.12066. 23859086

[clr70017-bib-0023] Naert, I. , G. Alsaadi , and M. Quirynen . 2004. “Prosthetic Aspects and Patient Satisfaction With Two‐Implant‐Retained Mandibular Overdentures: A 10‐Year Randomized Clinical Study.” International Journal of Prosthodontics 17: 401–410. 15382775

[clr70017-bib-0024] Ortega‐Oller, I. , F. Suárez , P. Galindo‐Moreno , et al. 2014. “The Influence of Implant Diameter on Its Survival: A Meta‐Analysis Based on Prospective Clinical Trials.” Journal of Periodontology 85, no. 4: 569–580. 23905841 10.1902/jop.2013.130043

[clr70017-bib-0025] Park, J. H. , S. W. Shin , and J. Y. Lee . 2023. “Narrow‐Diameter Versus Regular‐Diameter Dental Implants for Mandibular Overdentures: A Systematic Review and Meta‐Analysis.” Journal of Prosthodontics 32, no. 8: 669–678. 10.1111/jopr.13726. 37365991

[clr70017-bib-0026] Patil, P. G. , L. L. Seow , R. Uddanwadikar , and P. D. Ukey . 2021. “Biomechanical Behavior of Mandibular Overdenture Retained by Two Standard Implants or 2 Mini Implants: A 3‐Dimensional Finite Element Analysis.” Journal of Prosthetic Dentistry 125, no. 1: 138.e1–138.e8. 10.1016/j.prosdent.2020.09.015. 33393474

[clr70017-bib-0027] Pisani, M. X. , A. G. C. Presotto , M. F. Mesquita , V. A. R. Barão , D. T. Kemmoku , and A. A. del Bel Cury . 2018. “Biomechanical Behavior of 2‐Implant‐ and Single‐Implant‐Retained Mandibular Overdentures With Conventional or Mini Implants.” Journal of Prosthetic Dentistry 120, no. 3: 421–430. 10.1016/j.prosdent.2017.12.012. 29703669

[clr70017-bib-0028] Ravidà, A. , M. Tattan , H. Askar , S. Barootchi , L. Tavelli , and H.‐L. Wang . 2019. “Comparison of Three Different Types of Implant‐Supported Fixed Dental Prostheses: A Long‐Term Retrospective Study of Clinical Outcomes and Cost‐Effectiveness.” Clinical Oral Implants Research 30: 295–305. 10.1111/clr.13415. 30758878

[clr70017-bib-0029] Schiegnitz, E. , and B. Al‐Nawas . 2018. “Narrow‐Diameter Implants: A Systematic Review and Meta‐Analysis.” Clinical Oral Implants Research 29, no. Suppl 16: 21–40. 10.1111/clr.13272. 30328192

[clr70017-bib-0030] Schwindling, F. S. , and F. P. Schwindling . 2016. “Mini Dental Implants Retaining Mandibular Overdentures: A Dental Practice‐Based Retrospective Analysis.” Journal of Prosthodontic Research 60, no. 3: 193–198. 10.1016/j.jpor.2015.12.005. 26783089

[clr70017-bib-0031] Sohrabi, K. , A. Mushantat , S. Esfandiari , and J. Feine . 2012. “How Successful Are Small‐Diameter Implants? A Literature Review.” Clinical Oral Implants Research 23, no. 5: 515–525. 22313216 10.1111/j.1600-0501.2011.02410.x

[clr70017-bib-0032] Srinivasan, M. , P. Kamnoedboon , L. Angst , and F. Müller . 2023. “Oral Function in Completely Edentulous Patients Rehabilitated With Implant‐Supported Dental Prostheses: A Systematic Review and Meta‐Analysis.” Clinical Oral Implants Research 34, no. Suppl. 26: 196–239. 10.1111/clr.14068. 37750517

[clr70017-bib-0033] Zygogiannis, K. , I. H. Aartman , A. Parsa , A. Tahmaseb , and D. Wismeijer . 2017. “Implant Mandibular Overdentures Retained by Immediately Loaded Implants: A 1‐Year Randomized Trial Comparing the Clinical and Radiographic Outcomes Between Mini Dental Implants and Standard‐Sized Implants.” International Journal of Oral & Maxillofacial Implants 32, no. 6: 1377–1388. 10.11607/jomi.5981. 29140382

[clr70017-bib-0034] Zygogiannis, K. , D. Wismeijer , and A. Parsa . 2016. “A Pilot Study on Mandibular Overdentures Retained by Mini Dental Implants: Marginal Bone Level Changes and Patient‐Based Ratings of Clinical Outcome.” International Journal of Oral & Maxillofacial Implants 31, no. 5: 1171–1178. 10.11607/jomi.4339. 27632275

